# Intended actions and unexpected outcomes: automatic and controlled processing in a rapid motor task

**DOI:** 10.3389/fnhum.2012.00237

**Published:** 2012-08-16

**Authors:** Douglas O. Cheyne, Paul Ferrari, James A. Cheyne

**Affiliations:** ^1^Program in Neurosciences and Mental Health, Hospital for Sick Children Research InstituteToronto, ON, Canada; ^2^Department of Psychology, University of WaterlooWaterloo, ON, Canada

**Keywords:** automaticity, response inhibition, response switching, MEG, ERN, frontal theta, beta oscillations, motor cortex

## Abstract

Human action involves a combination of controlled and automatic behavior. These processes may interact in tasks requiring rapid response selection or inhibition, where temporal constraints preclude timely intervention by conscious, controlled processes over automatized prepotent responses. Such contexts tend to produce frequent errors, but also rapidly executed correct responses, both of which may sometimes be perceived as surprising, unintended, or “automatic”. In order to identify neural processes underlying these two aspects of cognitive control, we measured neuromagnetic brain activity in 12 right-handed subjects during manual responses to rapidly presented digits, with an infrequent target digit that required switching response hand (bimanual task) or response finger (unimanual task). Automaticity of responding was evidenced by response speeding (shorter response times) prior to both failed and fast correct switches. Consistent with this automaticity interpretation of fast correct switches, we observed bilateral motor preparation, as indexed by suppression of beta band (15–30 Hz) oscillations in motor cortex, prior to processing of the switch cue in the bimanual task. In contrast, right frontal theta activity (4–8 Hz) accompanying correct switch responses began after cue onset, suggesting that it reflected controlled inhibition of the default response. Further, this activity was reduced on fast correct switch trials suggesting a more automatic mode of inhibitory control. We also observed post-movement (event-related negativity) ERN-like responses and theta band increases in medial and anterior frontal regions that were significantly larger on error trials, and may reflect a combination of error and delayed inhibitory signals. We conclude that both automatic and controlled processes are engaged in parallel during rapid motor tasks, and that the relative strength and timing of these processes may underlie both optimal task performance and subjective experiences of automaticity or control.

## Introduction

Human action is a mix of controlled and automatic behavior and a remarkable proportion of human action appears to be of the latter sort (Bargh and Chartrand, 1999). Automatic actions are typically rapid, smooth, and cognitively effortless. They also require little or no explicit monitoring or conscious attention (Sloman, 1996; Evans, 2008; Kahneman, 2011). Conscious, controlled processes intervene, however, in tasks with complex rules or unexpected events. In repetitious automated activities, errors tend to increase when task constraints (e.g., a rapid succession of critical events requiring speeded responses) prevent intervention by controlled responses in a timely fashion or when distracting external events or interfering internal processes draw attention from the task at hand (Cheyne et al., 2011). One interpretation of such transient failures in cognitive control is that they reflect the occasional activation or intrusion of “default” brain states that interfere with the detection or processing of external cues (Mason et al., 2007; Eichele et al., 2008). Attention *lapses* are a common class of such interfering internal processes (Reason, 1999). An alternative, but not necessarily incompatible viewpoint, is that these failures in cognitive control may be induced by the tendency for our actions to become more automatized in such tasks, resulting in the simultaneous and sometimes conflicting engagement of cortical networks involved in preparation for a rapid motor response, and those involved in the conscious monitoring of cues that might indicate the need to rapidly change or withhold the response. The latter is exemplified by the tendency of the more frequent (default) movement or action to become “prepotent” leading to failures of inhibition as well as selection errors, and implies that neural events that underlie stimulus identification and those that underlie the preparation for movement occur in parallel, rather than as a sequential process. This competing process model may explain why subjects often make errors even when they fully attend to the task, and consciously detect the occurrence of the target stimulus during errors, as reflected by the typical absence of differences in sensory responses between correct and incorrect trials (although see Boehler et al., 2009). Preparation for inhibition, or response switching, can also be under a degree of automatic control, in parallel with the prepotent response. Hence, though errors may increase when controlled processes go offline, inhibition and rapid response switching may be observed. Models of response inhibition, such as the horse-race model (Logan and Cowan, 1984) have also proposed that the initiation of movement (go processes) and processes required to inhibit a movement once initiated, occur in parallel, with the resulting action the outcome of the race between the two processes. Although many studies focus on failures in rapid decision making, the preceding arguments also imply that automatic processes can lead to successful outcomes, and there has been recent evidence that automatic processes may in fact underlie to a large extent normal motor behavior that would otherwise be attributed to top–down executive control (McBride et al., 2012). Thus, rather than a clear separation between consciously intended actions that lapse into automatic (and therefore failed control) there is a subtle interplay between these processes that is largely driven by the temporal demands of the task. More specifically, in the context of speeded response inhibition tasks, the relative timing of automatic motor preparation and cue driven inhibitory processes determine behavioral outcome and should be reflected in the time course of brain activity in task specific brain networks, however, to date few neuroimaging studies that have been able to clearly separate these neural processes.

Brain activity accompanying cognitive control and response inhibition has been imaged with high spatial precision using hemodynamic imaging methods such as fMRI or PET (Ullsperger and von Cramon, 2001; Holroyd et al., 2004; Ridderinkhof et al., 2004; Aron and Poldrack, 2005; Garavan et al., 2006; Chevrier et al., 2007; Lutcke and Frahm, 2008; Kenner et al., 2010). These studies implicate a number of brain regions in the inhibition of motor responses, in particular, the right inferior frontal cortex (Garavan et al., 2006) which is thought to receive input from posterior sensory areas processing the stimulus cue and subsequently to generate inhibitory signals that propagate to cortical motor areas, or even more directly to subcortical motor structures such as the subthalamic nucleus (Aron et al., 2007) to cancel motor execution. Such studies have also confirmed the well-known role of medial frontal regions such as the dorsal anterior cingulate cortex (dACC) and supplementary motor area (SMA) in response selection and preparation (Jahanshahi et al., 1995; Paus, 2001; Cunnington et al., 2002) or even more directly in automatic modes of inhibitory control (Sumner et al., 2007). In the context of the dual-process or horse-race models we assume that preparatory signals from frontal midline areas and inhibitory signals from frontal brain regions impose competing go and inhibit signals on downstream motor areas, such as the primary motor cortex. Thus, the relative timing of such processes can determine successful inhibition, even in the case where an inhibitory stimulus is clearly perceived by the individual, who may consciously try, yet fail to inhibit an unwanted movement. However, hemodynamic measures such as fMRI and PET have limited temporal resolution making it difficult to distinguish brain activity involved in stages of early stimulus processing or response preparation from that related to response execution and inhibition. Similarly, for the analysis of error processing, these methods require caution in interpreting activations that may be related to rapid error detection or slower post-error behavioral adjustments (Chevrier and Schachar, 2010). These limitations highlight the need for techniques for measuring rapid event-related brain responses in order to reveal the time course of different neural processes underlying motor preparation, stimulus perception, and inhibitory control. There have been a number of recent studies involving go/no-go and other response inhibition tasks using both EEG (Carbonnell et al., 2004; Luu et al., 2004; Ridderinkhof et al., 2004; Schmajuk et al., 2006; O'Connell et al., 2007) and MEG techniques (Boehler et al., 2009; Mazaheri et al., 2009; Keil et al., 2010; Tzagarakis et al., 2010; Nigbur et al., 2012). MEG in particular offers the possibility of examining millisecond-by-millisecond cortical activity with high spatial accuracy when combined with advanced source reconstruction methods (Hillebrand and Barnes, 2005; Cheyne et al., 2006a). Most studies to date, however, have employed cued choice response tasks with long inter-stimulus intervals that preclude automaticity of responding, and tend to have relatively low error rates that make imaging error responses problematic.

Repetitive, monotonous tasks that are cognitively simple but attentionally demanding are paradigmatic occasions for attention lapse induced errors in the context of a highly prepotent response. One such task is the Sustained Attention to Response Task or SART (Robertson et al., 1997) requiring rapid responding to each of a sequentially presented (approximately 1 per second) quasi-random stream of single digits but periodically withholding responding to a selected target. SART performance has been related to self- and other-reported everyday attention lapses and to attentional problems in ADHD and TBI (Smilek et al., 2010). The structure of such tasks means that even the most transient lapses of attention to the immediate demands of the task rapidly lead to an increase in the likelihood of errors (Cheyne et al., 2006b, 2009a). Most response inhibition tasks, including the SART, involve complete withholding of a response to targets, which necessitates signal averaging relative to stimulus onset for successful inhibition trials, thereby emphasizing neural activity that is time-locked to processing of the stimulus, whereas movement preparation and error related activity is more robustly measured when time-locking to movement onset. A variant of the SART, the response switching task (Cheyne et al., 2009a) was recently applied in a study of the effect of attention lapses on the ability of subjects to switch responses to an infrequent target digit, rather than withhold their response altogether. This task produced similar error rates and response profiles to the SART, with concomitant experiences of frequent errors and unintentional responding. We chose this task for the current study as it allowed us to directly compare movement-locked neural processes during both error trials (default responses on target trials) that signify failed response inhibition and correct switch trials that reflect the successful inhibition of the prepotent default response. This task also allowed us to examine patterns of response speeding for fast and slow correct switch trials that might reflect the degree of automaticity in responding. We used a bimanual switch task to examine lateralized motor activity related to response inhibition. A subset of subjects performed the task in both directions to confirm that effects were independent of handedness and/or direction of switching. We compared this with a unimanual version of the switch task to control for differences that might be simply related to the bimanual aspects of the task. MEG measures were combined with advanced source modeling methods for both evoked and oscillatory brain activity to examine the timing of cortical activity related to response execution and inhibition, as well as the processing of error responses.

## Materials and methods

### Subjects

Fourteen healthy adults (five female, mean age 31.7 years, range 21–52 years) participated in this experiment. All subjects were recruited from the Toronto area and provided informed consent using protocols approved by the Hospital for Sick Children Research Ethics Board. The data from two participants were excluded due to poor task performance. All subjects were right-handed as assessed with the Edinburgh questionnaire (Oldfield, 1971).

### Response switching task

**Figure 1:**
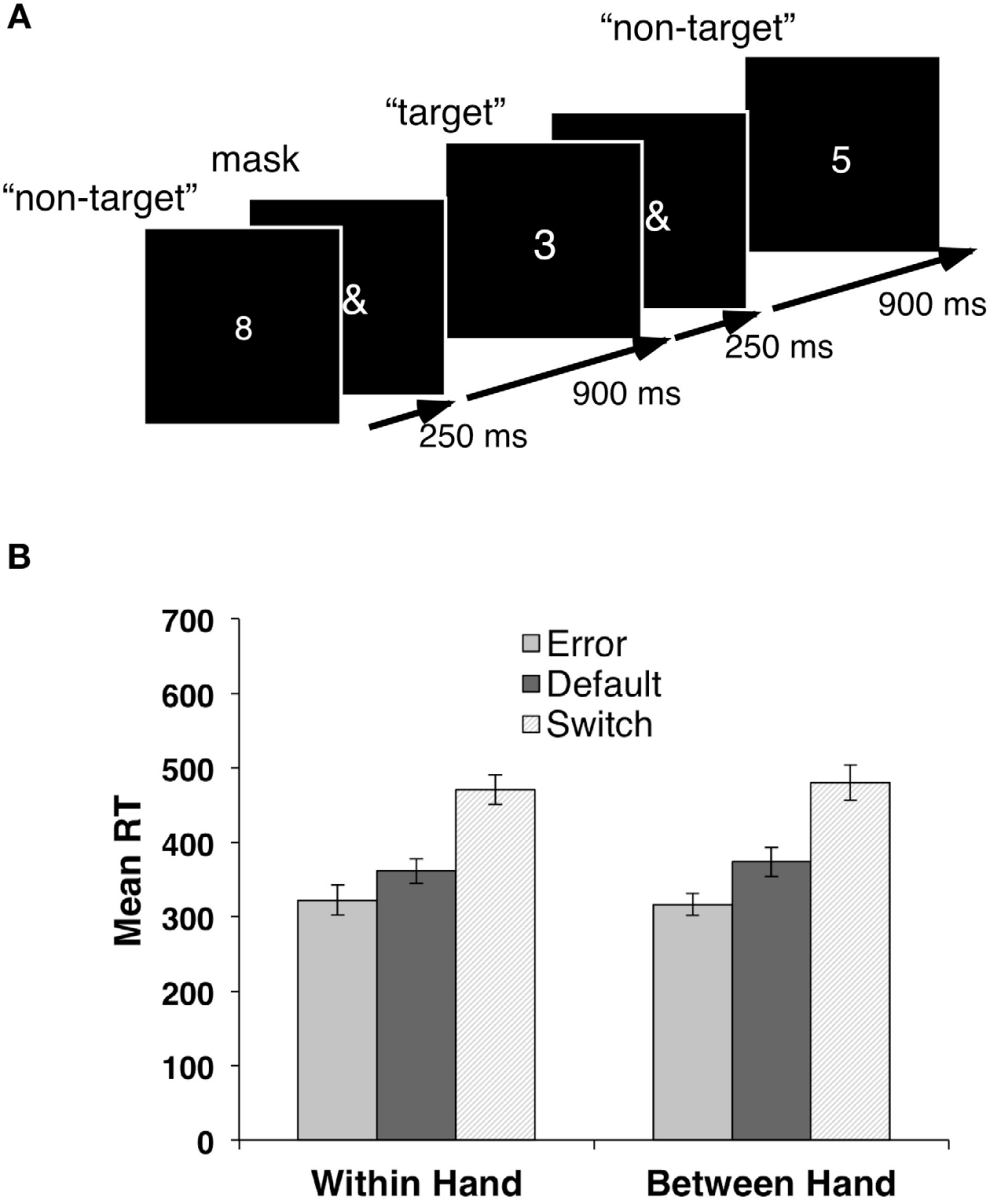
**(A)** Response switching task. Digits were presented every 1150 ms for a duration of 250 ms, followed by a stimulus mask (“&”) for 900 ms. Overall target digit (“3”) probability was 20%. **(B)** Response Time (RT) means and standard error for all trial types for the within-hand (WH) and between hand right-to-left (BH) tasks.

Each target stimulus (the digit “3”) was followed by an equal probability of 1, 3, 5, or 7 non-target stimuli (all other digits from “1” to “9”), resulting in an overall 20% probability of the occurrence of a target stimulus. Each digit was presented for a fixed duration of 250 ms with a constant inter-stimulus interval of 1150 ms, followed by presentation of a stimulus mask (“&” symbol) that remained on for 900 ms, until the presentation of the next digit (Figure 1A). No target stimulus immediately preceded or followed another target. Subjects performed two counterbalanced versions of the task; (1) a unimanual *Within-Hand (WH)* task required subjects to switch between fingers of their dominant (right) hand when presented with a target stimulus. The middle finger was the default response and the index finger the switch response for one-half of the subjects. This was reversed for the other half; (2) A bimanual *Between-hand (BH)* task for which the default movement was a button press with the right index finger with instructions to respond with the left index finger to the target stimulus (right-to-left switch). A subset of six subjects also performed the BH task reversed (i.e., left index finger was the default movement, left-to-right switch). Subjects were instructed to respond to the stimulus as quickly as possible but to avoid making errors.

### Behavioral measures

For all tasks we measured the number of incorrect (default) responses on target trials (Errors), mean Response Time (RT), and within subject response variability (RT SD) on default trials, as well as response speeding (reduced RT) on trials prior to targets.

### MEG recordings

Neuromagnetic activity was recorded using a whole head 151-channel CTF MEG system (MISL, Coquitlam, BC, Canada) in a magnetically shielded room. Data were collected at a sample rate of 625 samples/s, and a bandpass of 0–200 Hz. T1-weighted structural MR images were obtained from each subject using a Siemens 3T Magnetom Trio scanner. Small coils placed at fiducial locations (nasion and pre-auricular points) were used to monitor head position during recording and co-register source images to the subject's MRI. Subjects sat upright in an adjustable chair and motor responses were measured using a non-magnetic fiber optic response pad (LUMItouch Response System, Lightwave Medical Industries, Burnaby, Canada). Stimuli consisted of the digits 1–9 displayed in Times font with randomized font size with a visual angle of approximately 1.5–2.5 degrees presented via a LCD projector on a back-projection screen. For each task, a total of 1500 stimuli (1200 non-targets and 300 targets) were presented in a single recording lasting approximately 25 min.

### MEG analysis

For each task continuously recorded MEG data were segmented into epochs for each of four response types: (1) *default* movements which refer to correct movements of the default finger to the non-target stimuli, (2) *switch* movements where subjects correctly switched to the alternate response, (3) *errors* which refer to failures to switch (i.e., default movement is made to the target stimulus), and (4) *false alarms*, or switches made to non-target stimuli.

Localization of brain activity was carried out using both event-related and frequency based beamformer algorithms implemented in the *BrainWave* Matlab toolbox developed at the Hospital for Sick Children (http://cheynelab.utoronto.ca/BrainWaveSoftware2.html). An event-related beamformer method (Cheyne et al., 2006a, 2007) was used to generate source activity images for averaged brain responses from MEG recordings time-locked to movement onset. This is a spatial filtering method that computes volumetric images of instantaneous source power corresponding to selected time points in the averaged (evoked) brain responses. Data were first offline filtered from1 to 50 Hz for epochs extending from −1.5 s to 1.5 before and after movement onset. A time window of 1 s prior to 0.5 s following movement onset was used to compute the data covariance used in estimating the beamformer spatial filter weights from the single trial data. Beamformer images were created at 5 mm resolution every 5 ms over a period of 300 ms preceding to 300 ms following movement onset and images searched for peak activations. To select statistically significant peaks of activation across subjects, group averaging of functional images was carried out and thresholded using a non-parametric permutation test adapted for beamformer source images (Singh et al., 2003). In cases where strong activations (e.g., in the contralateral motor cortex) biased the omnibus permutation test (Chau et al., 2004) analysis was restricted to a region of interest anterior to the precentral sulcus. To detect differences in activity between conditions, contrast images were computed by subtracting source images for two different conditions (e.g., error minus control). Spatial normalization based on the MNI (T1) template brain was carried out using SPM8 (Welcome Institute of Cognitive Neurology, London, UK). Talairach coordinates of peaks activations were determined from the normalized images using the MNI to Talairach daemon (Lancaster et al., 2000). Statistically thresholded source images were superimposed on the MNI (ch2) template brain (Collins et al., 1994) using the mricron program (http://www.mccauslandcenter.sc.edu/mricro).

### Time-frequency analysis

In order to measure changes in induced cortical oscillations during response preparation and error detection, we used the synthetic aperture magnetometry (SAM) algorithm (Robinson and Vrba, 1999). Whole-brain pseudo-t difference images were created using by subtracting the source power during an active time window of 500 ms duration from a baseline period of equal duration in the theta (4–8 Hz), alpha (9–12 Hz), and beta (15–30 Hz) frequency bands. The baseline time window was set from −1.0 to −0.5 s preceding movement onset and the active time window shifted in 50 ms increments from the period immediately following the baseline window to the period following the motor response. Group averaging and permutation thresholds were applied in the same manner as described for the event-related analyses to detect peak locations of oscillatory changes. Time-frequency representations (TFRs) were constructed using a Morlet wavelet frequency transformation (Tallon-Baudry et al., 1997) of single trial source activity over a frequency range of 1–50 Hz in 1 Hz steps, and converted to percent change in power relative to a pre-movement baseline. For these TFR plots, a baseline from −0.8 to −0.6 s preceding button press was used in order to avoid overlap with the theta and alpha activations extending from the preceding response. In addition, in order to exclude the possibility that changes in oscillatory power were not simply reflecting the signal power of the strongly phase-locked motor fields accompanying movement onset, particularly at the lower theta and alpha frequency range, mean power was subtracted from the single trial power in the time-frequency plots to image primarily *induced* oscillatory activity. This is equivalent to removing the purely phase-locked activity (evoked response) from the data prior to time-frequency decomposition. A similar approach was used in an MEG study by Keil and colleagues (Keil et al., 2010) to image post-error frontal theta-band activity. This procedure allowed us to detect low-frequency theta bursts in frontal brain regions that would otherwise be overshadowed by signal power extending over the 1–12 Hz range due to the movement-evoked fields (MEFs) arising in primary motor cortex. It should be noted that some phase-locked activity will still remain due to trial-to-trial variability in timing, and similarly, some induced activity will have randomly consistent phase and thus also be suppressed by this procedure, such that it is difficult to clearly disentangle these two phenomena or verify whether evoked activity constitutes a phase-reset induced oscillation that becomes phase-locked to the movement. Detailed analyses of the relative contributions of phase-locked theta activity to post-error averaged responses in the EEG has been carried out by Makeig and colleagues (Luu et al., 2004; Makeig et al., 2004).

## Results

### Behavioral results

Behavioral results closely matched those of previous behavioral findings using this task (Cheyne et al., 2009a) and were highly similar for both unimanual and bimanual tasks, indicating that performance was independent of the specific motoric aspects of the task. Subjects made very few false alarms (switches to non-target stimuli) and these trials were not examined further.

#### Mean RT

A 2 (Switch Condition: Hand versus Finger) by 3 (Trial Type: Error, Default, and Switch) repeated measures ANOVA yielded a significant effect only for Trial Type [*F*_(2, 22)_ = 70.92, *p* < 0.001] for mean RT. Follow-up *t*-tests indicated that mean RT for each Trial Type differed significantly from each of the other two, all *p* values <0.01. Mean RT was greatest for switch trials and least for error trials (Figure 1B). Mean RTs across both conditions and all three response types were all significantly positively correlated, mean *r* = 0.61, *p* < 0.05, indicating the individual differences in response speed were consistent across conditions and trial types.

#### Switch trial RT and response speeding

**Figure 2:**
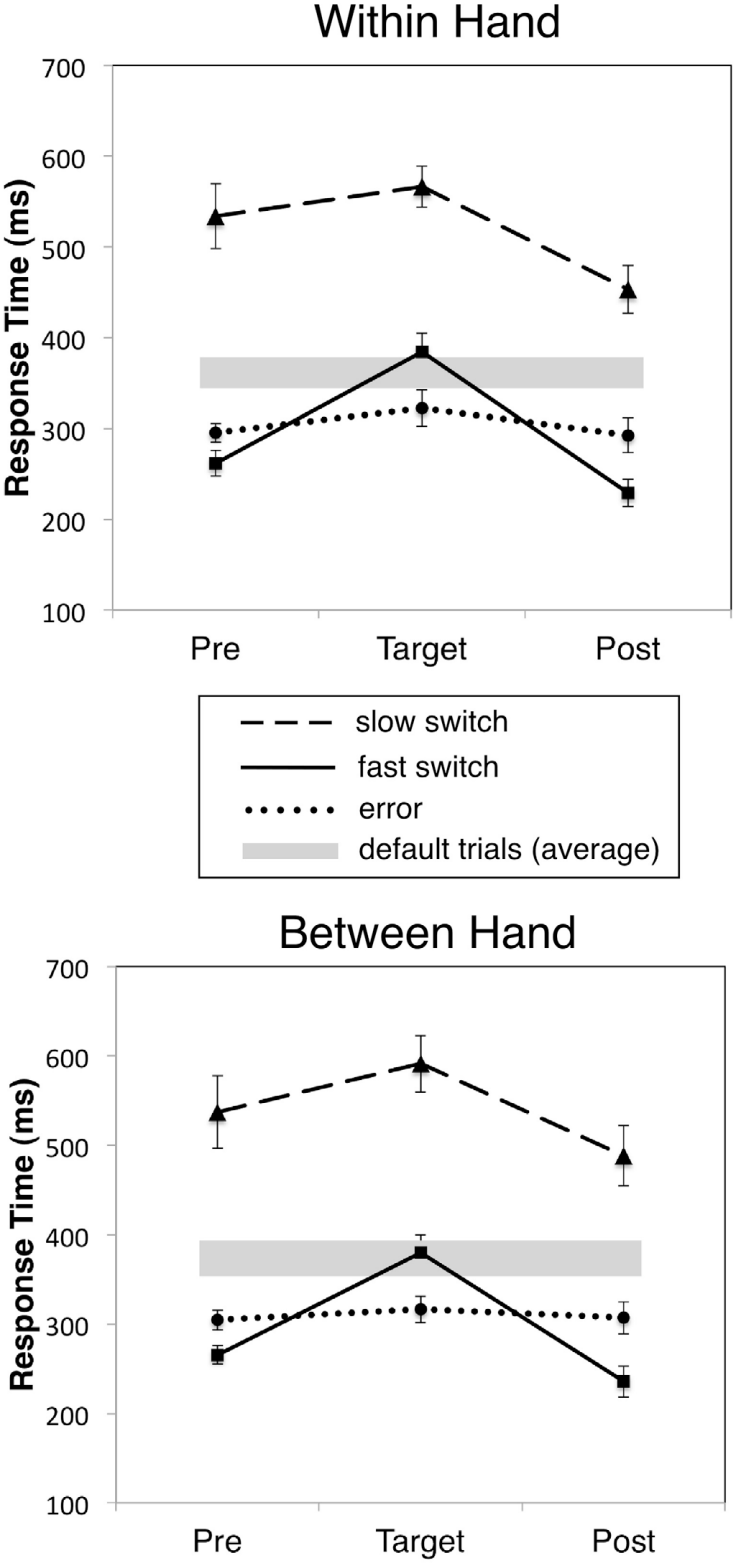
Mean RT for fast, slow correct and incorrect responses to target digits and the immediately preceding (Pre) and following (Post) defaults (non-target) responses for WH and BH tasks. Slow and fast switch trials correspond to the fastest 1/3 and slowest 1/3 RTs, respectively. Error bars indicate mean standard error, and gray bars the mean ±1.0 standard deviation RT for all default trials.

Our behavioral criterion for distinguishing automatic from controlled switch responses involved separating trials into subsets of “fast” and “slow” switch responses, defined as the shortest 1/3 and longest 1/3 switch RTs, respectively. Response speeding is well-known to accompany increased error rates in the SART and thus reflect “off-task” or more automated responding (Cheyne et al., 2006b). Surprisingly, we found that for the 1/3 fastest RTs for correct switches, the preceding trial showed an even faster RT than that preceding an error trial, and that this speeding continued in the trial following the correct switch response, even though RT for the switch response itself was still significantly slower than either error or default responses. Figure 2 compares the RTs for fast and slow correct switch and error trials, and the immediately preceding and following responses (which were always default responses to non-target stimuli) for both WH and BH conditions, showing response speeding (reduced RT) or response slowing (increased RT) immediately prior to or following the target response. For reference, the overall (grand mean) RT and standard error for all default trials is indicated by the gray bar. Results were strikingly similar across the two tasks. A 2 (Condition: Between Hand versus Within Hand) by 3 (Trial Position: Pre-target, Target, and Post-target) by 3 (Response Type: Slow Switch, Fast Switch, and Error) repeated measures ANOVA with RT as the dependent variable yielded significant Trial Position [*F*_(2, 22)_ = 161.41, *p* < 0.001], Response Type [*F*_(2, 22)_ = 101.65, *p* < 0.001], and Trial Position by Response Type effects [*F*_(4, 44)_ = 21.79, *p* < 0.001]. No effects involving Condition approached significance. The significant effect for Trial Position indicates that only switch trials were followed by a significant decrease in RT (post-switch speeding, *p* < 0.05). That is, error responses were associated with faster response speeds than both fast switch trials (and default responses) but were not associated with significant pre- or post-error speeding or slowing. Fast switch responses were, however, preceded and followed by significantly faster than pre-and post-errors responses in both tasks (*p* < 0.01).

#### Error rates

There was no significant difference in error rate between WH, mean = 29.31%, SD = 13.37 and BH conditions, mean = 28.06%, SD = 12.53, *t*(11) = 0.59, *p* = 0.57. For WH conditions there was no significant order difference in error rate between index-to-middle and middle-to-index fingers, *t*(10) = 0.95, *p* = 0.95. For BH conditions, error rate means were virtually identical, 30.28 (SD = 16.32) versus 30.93 (SD = 14.52), for right-to-left versus left-to-right respectively, *t*(5) = 0.22, *p* = 0.837. Mean error rates were comparable for all conditions. WH and BH error rates were robustly correlated, *r* = 0.90, *p* < 0.001. Overall, error means and individual differences in error rates were strikingly consistent across all three tasks.

### Event-related and oscillatory brain activity

#### Evidence for response inhibition in primary motor cortex

**Figure 3:**
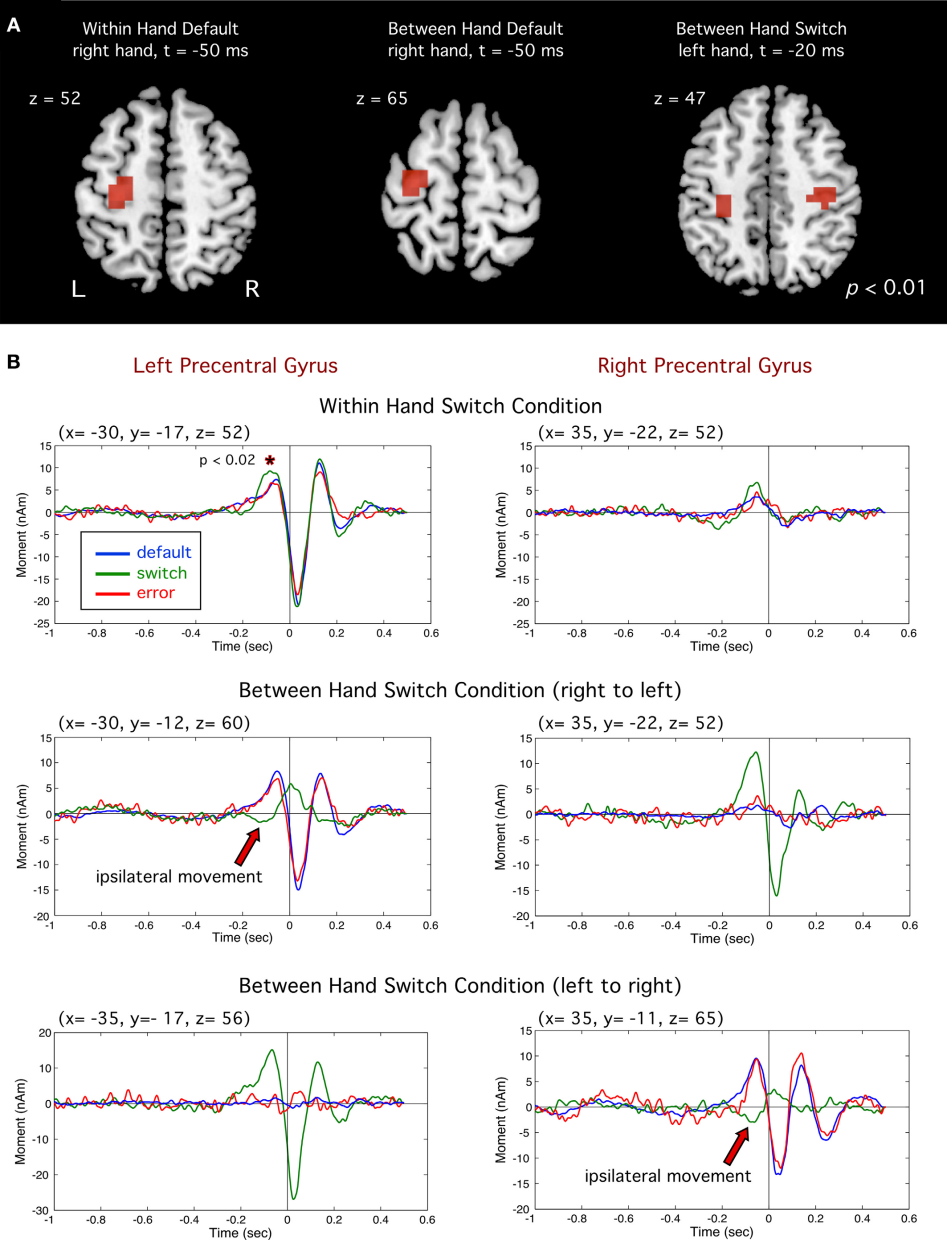
**(A)** Group images of event-related sources in motor cortex (precentral gyrus). Left and middle images show activity for default (right index) responses for the within hand (WH) and between hand (BH). Image at far right shows motor cortex activity for switch trials (left index finger). **(B)** Averaged source waveforms of brain activity in the left and right motor cortex for all conditions. For switch trials (green traces) significantly larger amplitude was observed prior to movement onset (^*^p < 0.02). Polarity reversals ipsilateral to the switch hand for successful switch trials are indicated by red arrows.

Event-related beamformer analysis revealed typical motor fields beginning approximately 200 ms prior to movement onset in the precentral gyrus (BA 4 and 6) in the primary motor cortex contralateral to the side of movement, reaching peak amplitude at approximately 50 ms prior to movement onset, followed by a MEF 30–50 ms after movement onset (Cheyne and Weinberg, 1989; Cheyne et al., 2006a). Figure 3A shows the source locations for significant voxels (*p* < 0.01) superimposed on a template brain. Grand averaged time courses (Figure 3B) revealed very similar motor fields for both default and error movements (i.e., movement of the same finger to both non-targets and targets). However, correct switch movements showed notable differences prior to movement onset. In the WH condition, a slight dip in the motor field is observed at around −200 ms, followed by a significant increase in peak amplitude at −100 ms (*p* < 0.02, paired *t*-test). This difference was significant for both middle-to-index and index-to-middle switching, and therefore not a bias of response finger. Moreover, in both BH conditions, the correct switch trials showed a complete polarity reversal of the motor field in the ipsilateral motor cortex (i.e., the motor cortex contralateral to the hand that had to be inhibited) at the same latency (red arrows in Figure 3B). The use of fixed source orientation for the beamformer calculations, and inspection of field patterns in the sensor data confirmed that this constituted a true reversal of current direction (dipole orientation). This ipsilateral activity corresponded to a significant ipsilateral peak in the source images for the switch movements (Figure 3A, right).

#### Evidence for controlled response switching: pre-movement frontal theta activity

**Figure 4:**
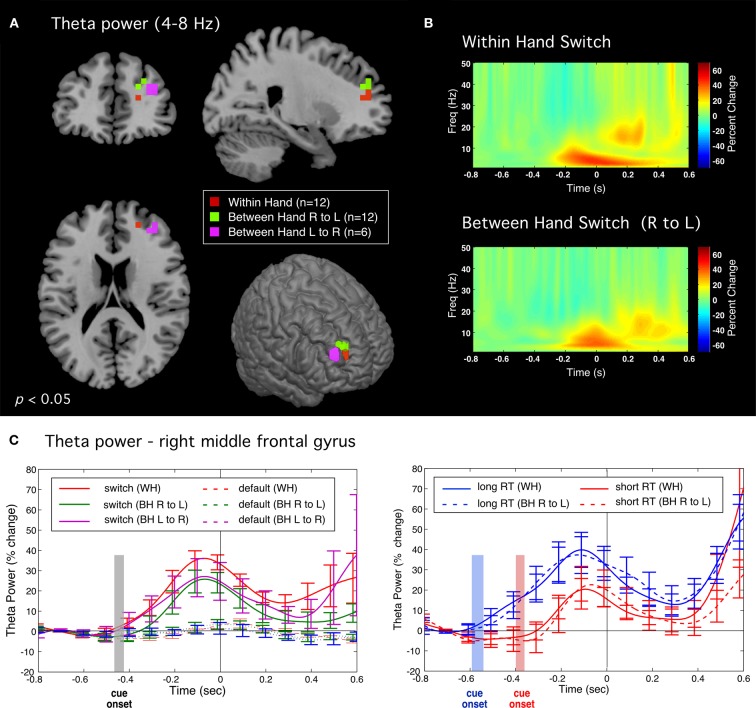
**Theta band (4–8 Hz) activity preceding correct switch responses. (A)** Significant activations in the right middle frontal gyrus for all tasks during the pre-movement period (−0.5 to 0 s) relative to baseline (−1 to −0.5 s). **(B)** Time-frequency plots of induced source activity (1–50 Hz) for the peak locations shown in **(A)** for the within hand (WH) and between hand (right to left) conditions. **(C)** Time course of total power within the theta (4–8 Hz) band comparing switch and default trials (left plot). Plot on right shows time courses for subsets of “fast” and “slow” switch trials for both WH condition (solid traces) and BH conditions (dotted traces). Shaded bars indicate the time range of stimulus onset (mean ± standard error) for the WH condition.

Differential power images using the SAM beamformer algorithm revealed a transient increase in theta band (4–8 Hz) activity preceding movement onset for correct switch movements for all tasks. This activity was localized to similar locations in the right middle frontal gyrus (BA 10) for both unimanual and in the bimanual tasks (Figure 4A), independent of the side of movement (mean Talairach coordinates across tasks: *x* = 23, *y* = 46, *z* = 18). Theta band time course was similar across all tasks (Figure 4B) beginning shortly after the stimulus cue onset (−200 ms) and reaching maximum power at around 100 ms prior to movement onset, and significantly greater than in default trials (*p* < 0.005, paired *t*-test, corrected), which showed little to no activation in this brain region. Figure 4C shows theta time course for subsets of fast and slow switch trials for both WH and BH conditions. A two-way repeated measures ANOVA of RT versus task indicated that theta onset time (latency at which theta power began to increase above baseline) was significantly later for fast RT trials [*F*_(1, 11)_ = 6.10, *p* < 0.031] and total theta power (area under the curve) was reduced during the pre-movement period (−800 to 0 ms) for fast RT trials [*F*_(1, 11)_ = 6.10, *p* < 0.031]. This association of theta power with slow switches and its reduction during fast switches is consistent with a role for right middle frontal theta in controlled processes. There was no significant main effect for task as can be seen by the similarity in the theta activity time profiles for WH and BH conditions.

#### Evidence for automatic response preparation: beta suppression in motor cortex

**Figure 5:**
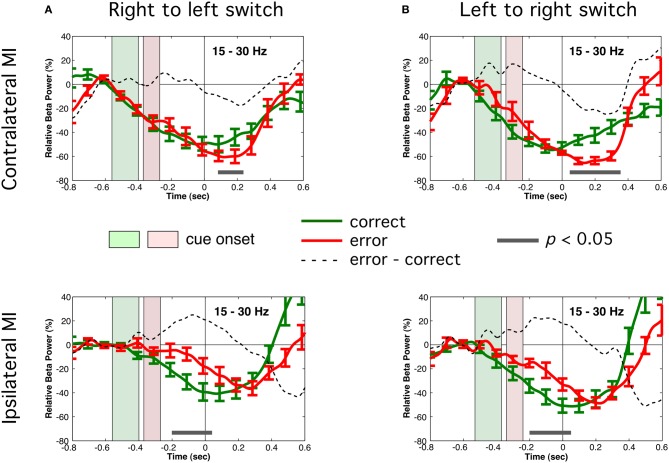
**Time course of total power in the beta (15–30 Hz) band in motor cortex in both bimanual tasks.** Contralateral motor cortex (upper row) and ipsilateral MI (lower row) correspond to motor cortex contralateral and ipsilateral to the default response hand, respectively. Correct trials are shown in green and error trials are shown in red. Shaded rectangles show the time range (±1.0 SD around the mean) of visual cue onset for the correct (green shading) and error (red shading) trials. Dotted lines show the difference (error–correct) in beta power. **(A)** Right to left switch condition (contralateral MI = left motor cortex). **(B)** Left to right switch condition (contralateral = right motor cortex). Solid grey lines indicate period during which differences between correct and error trials was significant (paired *t*-test, *p* < 0.05).

In all tasks and trial types, a robust pre-movement suppression of beta band (15–30 Hz) activity was observed in primary motor cortex. Figure 5 shows the time course of relative beta band power (baseline −700 to −500 ms) for correct switches and errors for the bimanual tasks for the motor cortex contralateral or ipsilateral to the default hand. It can be seen that beta suppression began very early in all cases, around 600 ms prior to movement onset and preceded presentation of the cue (indicated by shaded area corresponding to mean RT ± 1.0 standard deviation) reaching maximal values of about 60% reduction in power relative to the pre-cue baseline. The pattern of beta suppression was highly similar for both directions of switching (right to left or left to right). A three-way repeated measures ANOVA of beta suppression for the BH right-to-left condition comparing hemisphere (contralateral or ipsilateral), response type (correct switch or error) and time (−300, −200, −100, and 0 ms preceding movement) revealed a main effect for hemisphere for both right to left [*F*_(1, 11)_ = 19.71, *p* < 0.001] and left to right switches [*F*_(1, 11)_ = 19.24, *p* < 0.007]. Thus, beta suppression was largest contralateral to the side of the default (prepotent) movement, independently of the side of movement. However, beta suppression in the motor cortex ipsilateral to the default hand was reduced and delayed on error trials, as revealed by a significant hemisphere by trial type interaction [*F*_(3, 33)_ = 18.91, *p* < 0.001 for within hand). This difference was significant from −200 to 50 ms relative to movement onset (grey line in Figure 5, lower). Thus, beta suppression was more lateralized on errors at the time of stimulus processing, and remained so until the motor response was executed. In the motor cortex contralateral to the default hand, beta suppression began to return to baseline more rapidly on correct switch trials beginning around movement onset (Figure 5, upper) confirmed by a significant three-way interaction for hemisphere, trial type and time [*F*_(3, 33)_ = 5.34, *p* < 0.012]. These effects where replicated left to right switch data [*F*_(3, 33)_ = 18.91, *p* < 0.001 for Hemisphere by Trial Type interaction] and three-way interaction [*F*_(3, 15)_ = 3.71, *p* < 0.035]. The similarity between both bimanual tasks was further confirmed by collapsing across tasks (*n* = 6) with no significant main effects (all *F* values <1.0). In contrast to the bimanual tasks, beta suppression for the within hand (WH) task also began early with a similar time course, but showed largely contralateral suppression and a delayed or very weak ipsilateral beta suppression (data not shown).

#### Error-related responses in dorsal ACC

**Figure 6:**
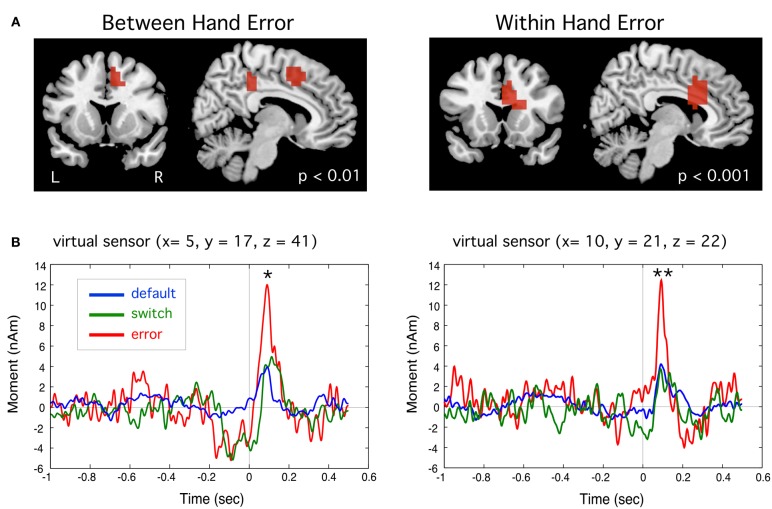
**(A)** Event-related beamformer source images showing error-related brain activation a latency of approximately 90 ms following the incorrect button press in the region of the anterior cingulate cortex for between hand (right-to-left) and within hand switch tasks. **(B)** Group averaged time course of source activity relative to movement onset (*t* = 0 s) for peak activations shown. Peak activity for error trials was significantly different from default trials for both tasks (^*^p < 0.01, ^**^p< 0.001, paired *t*-tests, corrected).

Event-related source images revealed a statistically significant transient peak of activity occurring around 90 ms following the onset of an error movement localized to the region of the dACC of the right hemisphere (BA 32) in the BH condition, and slightly more inferior in the WH condition (BA 24), as shown in Figure 6. This peak was brief in duration, but significantly larger than small activations at the same latency for switch and default trials in both WH and BH conditions (*p* < 0.01 and *p* < 0.001, paired *t*-tests, corrected). Importantly, this dACC activity reached maximum amplitude roughly during the zero crossing of activity in MI and is thus unlikely to reflect cross-talk from the larger MEF responses which peaked around 40 and 150 ms, respectively.

#### Error-related frontal theta activity

**Figure 7:**
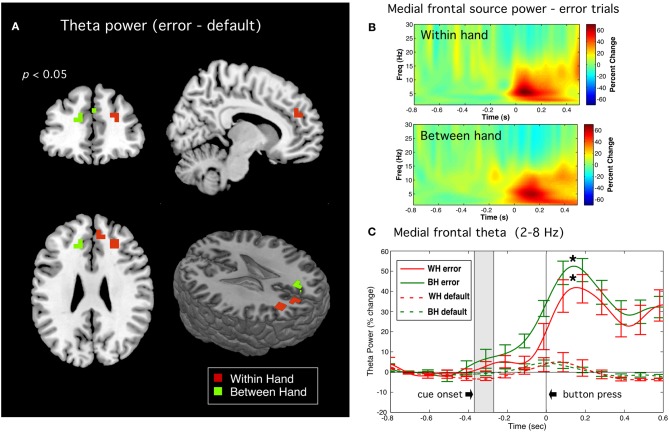
**Error related theta band activity. (A)** Source images of theta (2–8 Hz) power (*p* < 0.05) for the Error > Default contrast for within hand (WH) and between hand (BH) tasks. **(B)** Time-frequency plots for virtual sensors corresponding to the peak locations. **(C)** Time course of total power in the theta band for error trials (solid lines) and default trials (dotted lines) for both WH and BH conditions. Shaded rectangles indicate the approximate time range (±1.0 standard deviation from the mean) of visual cue onset. Vertical bars indicate standard error across subjects. Difference in peak theta power at latency of 150 ms following button press (indicated by asterisks) between error and default trials were highly significant for both WH and BH conditions (*p* < 0.0004 and *p* < 0.00003, respectively, paired *t*-tests, corrected).

Significant peaks of theta band activity were observed following onset of error responses in anterior regions of the frontal midline, including medial frontal gyrus and anterior cingulate cortex (ACC). A bandpass of 2–8 Hz was used as time-frequency plots revealed this activity to have significant power at frequency down to 4 Hz. Threshholded contrast images (*p* < 0.05) between error and default trial types (Error > Default) are shown in Figure 7A, revealing peaks in the region of ACC and medial frontal gyrus in both left and right hemisphere. Figure 7B displays the time-frequency plots of source activity (virtual sensors) for these peak locations showing highly robust theta bursts following movement onset on error trials in both tasks. Figure 7C displays the time course of total power of theta band activity (i.e., averaged across the theta frequency band) showing that the observed theta increase was highly significantly different from default trials (*p* < 0.0005, paired *t*-tests, corrected). Similar results were observed on error trials for the subset of subject for the left to right switch task but for brevity only the tasks involving all 12 subjects are shown.

## Discussion

The present study revealed a number of cortical events and oscillatory activity patterns associated with inhibition, response switching, and errors in a continuous performance response switching task. In particular, the locations and timing of these cortical events and activities were consistent with the theoretical properties of automatic (rapid, habitual, and prepotent) and controlled (slow, rule-based, and occasional) response modes. Specific evidence for these effects and alternative interpretations are considered in the following sections.

### Response switching and inhibitory control

Correct switches involved suppression of a highly prepotent default response, as reflected by relatively high error rates (~30%) in this task. Consistent with this claim, we observed consistent transient changes in primary motor cortex, as well as changes in oscillatory brain activity in frontal regions when subjects successfully switched response hands or fingers to target stimuli. A novel observation in this study was a transient change in primary motor cortex around 200 ms prior to onset of a correct switch movement contralateral to the hand that was inhibited (Figure 3). In the unimanual task, this was observed as a small, yet significant, amplitude increase. In the bimanual task, however, this can be seen as a complete reversal in source waveform polarity, indicating intracellular currents with opposite orientation. The pre-movement motor field reflects anterior directed dipole sources in the precentral gyrus (Cheyne and Weinberg, 1989). Since MEG is thought to reflect intracellular currents in tangentially oriented pyramidal cells, this likely reflects excitatory input to superficial cortical layers in the anterior wall of the central sulcus, resulting in anterior directed intracellular currents and surface negative extracellular current flow that generates the readiness potential in the EEG, and is thought to be due to excitatory input to the apical dendrites of pyramidal cells in area 4 (Ikeda and Shibasaki, 1992). Although speculative, the observed polarity reversal in ipsilateral MI might be attributed to a shift from excitatory to inhibitory synaptic input to the same cortical layers in MI, directly inhibiting motor output related to the default response. However, we cannot rule out the activation of different populations of neurons in nearby cortical areas such as excitatory input in the precentral or postcentral sulcus. These findings corroborate previous reports of a positive pre-movement potential recorded from electrodes overlying the motor cortex contralateral to the inhibited side of movement in speeded between-hand choice tasks (Vidal et al., 2003; Carbonnell et al., 2004) that were also interpreted as reflecting inhibition of the non-selected movement. To our knowledge, our findings represent the first source analysis results showing a dipole polarity reversal localized to the region of MI associated with inhibition of a motor response. Moreover, a change in the motor field at a similar latency in the motor cortex contralateral to both the inhibited and moving finger in the unimanual task, lends further evidence that these changes may reflect the downstream effect of inhibition at the level of the primary motor cortex. Importantly, no change was detected at this latency in error trials, indicating that it is specific to successful response inhibition.

Further upstream we observed a transient increase in theta band (4–8 Hz) activity only for correct switch trials. This activity was lateralized to an anterior region of the right middle frontal gyrus in all tasks, independently of response hand or whether inhibition was bimanual or unimanual. As noted, this brain region is highly implicated in response inhibition (Aron, 2007; Chevrier et al., 2007; Isoda and Hikosaka, 2007; Picton et al., 2007; Swann et al., 2009) and we interpret these findings as most likely reflecting the active engagement of inhibitory brain mechanisms. Most importantly, switch related theta activity did not begin until after cue onset, as further confirmed by its delayed onset in trials with shorter RTs (Figure 4C), but in all conditions reached maximum power prior to movement onset, consistent with the active inhibition of motor output by engagement of control processes associated with frontal brain networks. Moreover, frontal theta activity was significantly reduced in amplitude for faster, and hence presumably automatic, switch responses suggesting a direct role in the strength of inhibitory control. Frontal theta oscillations have, however, been implicated in response-selection mapping or updating of tasks requirements within working memory (Womelsdorf et al., 2010). Given that correct responses in our task involved both inhibition of a default response and selection of an alternative response, we cannot rule out that this activity may be related to remapping of motor output on switch trials, although previous studies utilizing “partial” inhibition tasks have also reported right frontal activations (Coxon et al., 2009). Interestingly, there were no discernable differences in either the timing or amplitude of the ipsilateral motor fields described above for correct switch trials when analyzed separately for fast and slow switches, indicating that, unlike frontal theta activity, motor cortex changes were not sensitive to the speed of responding and may reflect an inhibition process further downstream and more tightly coupled to motor output during the switch response. Further studies are required to differentiate brain activity related to response selection from that related to response inhibition in tasks that involve an alternative response.

### Early beta band suppression and response preparation

We observed an early and steady suppression of beta band activity in motor cortex that began before processing of the stimulus cue, indicating that motor cortex activation begins immediately after completion of the previous response (Figure 5). It is well known that sensorimotor beta rhythms are strongly modulated during voluntary and cued motor responses (Pfurtscheller et al., 1996; Alegre et al., 2006; Jurkiewicz et al., 2006). In cued tasks, reduction in beta power typically begins following presentation of an imperative “go” cue, and may return to baseline on inhibited or withhold trials in go/no-go tasks (Zhang et al., 2008; Swann et al., 2009). The present findings are consistent with previous EEG studies showing that beta suppression can precede go cues when they are predictable (Alegre et al., 2003; Kilner et al., 2005). Interestingly, in our bimanual tasks, this early preparation was observed bilaterally in MI on correct switch trials, but was delayed and weaker in the MI contralateral to the switch hand on error trials. This was replicated for both right-to-left and left-to-right versions of the task in the same individuals indicating that this was not a handedness or hemispheric laterality effect. If beta suppression reflects motor preparation, this would suggest that there was automatic preparation of *both* responses prior to presentation of the response cue, but when this ipsilateral preparation was delayed or weaker, there was a bias towards failing to switch to the response hand. Our findings are consistent with the interpretation of beta suppression in motor cortex constituting a bias or threshold for initiating a response in the corresponding hand. Other studies have shown increased beta suppression when the response hand or movement parameters are known in advance of a go cue (Doyle et al., 2005; van Wijk et al., 2009; Tzagarakis et al., 2010), when speed is emphasized over accurate responses (Pastötter et al., 2012) or as a decision to move the left or right hand increases over the pre-movement period (Donner et al., 2009). Accordingly, in our right-handed unimanual task, beta suppression was lateralized to the left hemisphere, with weaker and delayed suppression in right motor cortex, consistent with the lack of need to prepare left hand responses.

It has been hypothesized that raised levels of beta oscillations in basal ganglia thalamo-cortical networks are “anti-kinetic” and that their suppression is necessary for motor readiness or the initiation of novel movements (Jenkinson and Brown, 2011). This claim is supported by observations of increased beta activity in these structures in disorders of movement initiation such as Parkinson's disease or dystonia (Brown, 2007; Crowell et al., 2012). Our findings are also compatible with a “disinhibition” hypothesis of motor cortex beta oscillations, in that the requirement for fast responding necessitates rapid suppression of beta activity bilaterally for successful bimanual switching, even prior to receiving the response cue. Thus, higher levels of beta power contralateral to the switch hand may have penalized successful initiation of the switch response leading to errors. An alternative interpretation is that beta modulation indicates shifts in attention to the response hand, although, such effects are usually found in the sensorimotor alpha or mu (9–12 Hz) band (Jones et al., 2010), and beta band lateralization has been shown to be influenced more by side of movement than lateralized spatial attention (Doyle et al., 2005). Although, we did not examine mu band activity in detail, it generally demonstrated a very different temporal pattern of activity in comparison to beta band activity and warrants further study.

### Error related activity in medial frontal cortex

We observed two types of brain activity related to response errors. The first was a brief transient response localized to dorsal regions of the anterior cingulate cortex (dACC) around 90 ms following onset of an error response (Figure 6), which is likely the magnetic counterpart to the electrically recorded event-related negativity (ERN) response (Gehring et al., 1993) that to date has only been reported in a few MEG studies (Miltner et al., 2003; Mazaheri et al., 2009). This putative ERNm response localized primarily to the right hemisphere independently of the side of movement, and was only detectable in the group averaged event-related beamformer virtual sensors when taking into account variable location and dipole polarity across subjects, which may explain why this response has been difficult to identify in previous MEG studies (Miltner et al., 2003; Stemmer et al., 2004). The anatomical location of our ERNm, although slightly different in the BH and WH tasks, is consistent with activation of cingulate motor areas such as the rostral cingulate zone, which has been implicated in both internal selection of alternative responses and unconscious monitoring the consequences of the selected action (Paus et al., 1993; Picard and Strick, 2001; Swainson et al., 2003). The dorsal ACC has also been suggested as the generator of the ERN as a brain region for detecting mismatch between planned action and outcome (Falkenstein et al., 1990; Dehaene et al., 1994; Vidal et al., 2000, 2003; Ridderinkhof et al., 2004). The latency of ERNm supports the hypothesis that it reflects a rapid, automatically elicited error signal due to mismatch between an efference copy of the motor plan and the executed response, rather than processing of sensory or proprioceptive feedback (Gehring et al., 1993; Vocat et al., 2008).

Second, we detected increased theta band activity immediately following movement onset from more anterior portions of the frontal midline with bilateral or mixed lateralization across tasks and subjects. This is consistent with reports of bilateral and widespread activation of regions of the frontal mesial cortex during errors in choice response tasks using fMRI (Lutcke and Frahm, 2008) and EEG (Luu et al., 2004; O'Connell et al., 2007; Nigbur et al., 2012). In contrast to the ERNm, theta activity in frontal regions was observed only on error trials (Figure 7C) indicating that it was specific to error detection. This activity also involved more widespread and anterior frontal regions and reached peak amplitude later than the ERNm, consistent with speculation that frontal theta rhythms reflect the processing of detected errors (Mazaheri et al., 2009; Keil et al., 2010; Nigbur et al., 2012).

Although, the ERNm response was significantly larger in error trials, small increases in activity at similar latencies could be seen in both the switch and default trials (Figure 6B). In addition, frontal theta increases began slightly prior to the button press and included peaks in right frontal regions observed in the successful switch trials (Figure 7C). This suggests that these brain regions may have been activated during both correct and incorrect responses, consistent with the putative role of the dorsal ACC in monitoring conflicting response tendencies (Carter et al., 1998; Botvinick et al., 2001; Bates et al., 2002; Hajcak et al., 2005). An alternative interpretation is that this activity is the consequence of an appropriate task-related decision that comes too late on error trials (Cheyne et al., 2009a). Isoda and Hikosaka (2007) observed delayed inhibitory signals in the premotor cortex of rhesus monkeys performing a cue-switching task, where successful performance was associated with activation of pre-SMA neurons, but only if this activity preceded response (saccade) initiation, providing evidence that delayed activity in this brain region represented a “switch signal that was unable to accomplish successful switching” (p. 241). Taken together with the fact that subjects were preparing their responses early and exhibited response speeding during error trials, our results suggests that some errors may have been associated with premotor activity that was too delayed or below threshold for successful switching during fast responses.

It should be noted that there was a complete absence of post-movement frontal theta activity on correct switches to targets. This provides evidence against the possibility that post-error theta oscillations simply reflect a stimulus-evoked “oddball” response to the infrequently presented target stimulus in the SART (Mazaheri et al., 2009). This also demonstrates the advantage of using a response switching task over go/no-go tasks for studying error-related motor responses, as the absence of a motor response on correct trials in the latter makes it difficult to directly compare successful and unsuccessful response inhibition to target stimuli.

### Intended actions and unexpected outcomes

#### The loss of agency

The present results are relevant to issues of the phenomenology of agency and its loss during self-initiated behavior. During response speeding subjects in our experiments often made errors that felt “unintended” as they experienced correctly detecting the switch cue while failing to inhibit the default response. Such a subjective experience would follow from experiencing the near-simultaneity of a (tardy) decision to switch and a failure to do so. During the response switching task, subjects report not only a loss of the sense of agency during errors but also a transfer of the sense of agency to the offending hand in an apparent attention-lapse alienation of agency (Cheyne et al., 2009a). We have previously noted similarities between this experience and alienation of the sense of agency in the “anarchic hand sign” (Della Sala et al., 1994; Eilan and Roessler, 2003) as well as independently derived neurocognitive theories for absent-minded actions (Della Sala et al., 1994; Eilan and Roessler, 2003; Smallwood and Schooler, 2006) as both have been discussed as failures of executive control over automatic processes. Such observations led to the speculation that, whereas the feelings of alienation of agency in anarchic hand sign were caused by damage to medial brain structures, similar feelings of loss of agency may arise in the switching task because of the temporal constraints inherent in the task. Hence, during errors the subject is left with the experience of observing himself perform an action that he was simultaneously countermanding.

#### Where did i go right?

An intriguing observation, which may represent the reciprocal of the error-induced alienation of agency, was that subjects would often relate the subjective experience of less “effort” or a sense of automaticity of some of their correct responses. As noted, right frontal pre-movement theta activity was reduced in amplitude for fast switch responses, which suggests less conscious effort or top–down control needed to inhibit the default movement on fast switch trials. This implies a somewhat paradoxical effect of lowered awareness on some successful switch trials—yet correctly responding as if the response selection system was on “autopilot.” Consistent with this interpretation was the further observation that, although fast switches were slower than mean error responses, pre-correct speeding of fast switches exceeded even pre-error RTs. Given that commonly employed behavioral markers of attention lapses in the SART are speeded RTs for go (default) trials prior to errors and slower RTs prior to successful inhibitions or switches (Robertson et al., 1997; Manly et al., 1999; Cheyne et al., 2009b), the finding that that responses on trials preceding fast switches were actually significantly faster than those preceding errors suggests that response speeding by itself does not necessarily determine that an error will be made, but rather that automatic processes are dominant, for better or worse. Interestingly, on these speeded correct trials, subjects did slow their responses significantly to perform the switch movement, yet resumed rapid responding on the subsequent trial. The immediate resumption of speeded responding following the fast switch is consistent with the hypothesis that automatic processing was maintained throughout the entire period preceding and following the target trial.

## Conclusions

Using advanced brain imaging techniques we were able to track the time course of neural processes in frontal and motor regions that reflected the conscious and controlled inhibition of prepotent default responses and selection of alternate responses to an infrequent switch cue. We observed processes that reflected automatic response preparation that likely enabled the subject's ability to perform the task at rapid rates, presumably under conditions of inconsistent or unstable conscious control. The latter was reflected by successful response switching during periods of highly speeded motor responses typically associated with higher error rates, and accompanied by a paradoxical experience of automaticity. Errors were nonetheless more likely during this faster and presumably more automated mode of responding, and also associated with the experience of an action that was not intended. This suggests that automaticity of our actions can have both positive and negative effects on performance and in both cases, may correspond to an altered sense of agency.

It is interesting to speculate that the simultaneous engagement of brain networks underlying different aspects of cognitive control, driven by the temporal demands of rapid responding, result in the subject fluctuating between automatic and controlled modes of responding associated, respectively, with the subjective experience of unexpected outcomes (good or bad), or simply actions as (implicitly) intended. We do not imply that changes in vigilance or attention lapses did not play a role in task performance. Previous EEG and MEG studies have provided evidence of decreased processing of sensory input attributable to attention lapses or mind wandering during the SART (Smallwood et al., 2008; Mazaheri et al., 2009), although in the current study, subjects were always aware of their errors and rarely switched on default trials, and inspection of visual responses to the cues did not reveal any consistent amplitude or latency changes across response types. Rather, attentional processes likely underlie the shifting between controlled and automatic modes of responding and changes in response speed may reflect changes in the attentional state of the subject, the neural correlates of which require further investigation. Indeed, the ability to carry out many skilled motor tasks, particularly rapidly executed ones, is associated with decreasing, rather than increasing conscious control over one's movements, a well-known phenomenon in athletics and other highly trained motor skills. Thus, it should not be surprising that optimal performance on speeded response tasks, such as the SART and response switching task is accompanied by a similar feeling of “flow” (Csikszentmihalyi, 1990) or being “in the zone.” Further investigation of how these aspects of action planning and inhibitory control relate to attentional mechanisms may help elucidate how automatic and controlled processes interact under conditions of high vigilance or attentional demand, as well as how these processes may be altered in populations with cognitive deficits.

### Conflict of interest statement

The authors declare that the research was conducted in the absence of any commercial or financial relationships that could be construed as a potential conflict of interest.
